# Supplementary material for: Investigation of the Hepatitis-B Vaccine’s Immune Response in a Non-Alcoholic Fatty Liver Disease Mouse Model
Source: Vaccines (Basel). 2024 Aug 22;12(8):934. doi: 10.3390/vaccines12080934 (PMC11359425; doi:10.3390/vaccines12080934)
Supplement: Supplementary file 1 [file vaccines-12-00934-s001.zip › vaccines-3069963-supplementary.pdf]

Investigation Of Hepatitis-B Vaccine Immunity In Non-Alcoholic Fatty Liver Disease Mouse Model

Supplementary file

Table S1. The formulations of experimental diets

Contents	HFD/CD Diet, %	Control Diet, %
Casein	20	20.96
Maltodextrin	10	10
Corn Starch	19.86	45
Sucrose	16	7
Cellulose	5	5.6
Soybean oil	8.59	3.15
Beef tallow	15	-
Sepiolite	0.68	1
L-cystine	0.37	0.5
Choline bitartrate	-	0.19
Vit-Mix*	1	1.8
Min-Mix**1	3.5	4.8

*AIN-93G Vitamin mix **AIN-93M Mineral mix

Table S2. Hepatitis B Vaccination Programs Applied in Control and NAFLD Groups.

	1. Vaccination	2. Vaccination	3. Vaccination
Day	0	7	21
ND	4 µg	4 µg	4 µg
HD2	8 µg	8 µg	-
HD3	8 µg	8 µg	8 µg

Table S3. Pre-vaccination period (Week 0-10 week) body weights in the experimental groups.*

	Control groups	NAFLD groups	P**
N	18	21	
Day 0	17.2 (16.9-18.6)	17.7 (17.4-18.1)	0.165
Week 1	18.7 (17.9-19.8)	20.6 (20.3-21.3)	<0.001
Week 2	19.8 (19.4-22.1)	21.7 (21.4-23.0)	<0.001
Week 4	21.5 (20.4-23.5)	25.2 (24.1-26.3)	<0.001
Week 6	22.5 (21.4-25.1)	27.6 (25.5-29.9)	<0.001
Week 8	22.8 (22.4-25.9)	28.8 (26.8-33.1)	<0.001
Week 10	25.9 (25.2-27.3)	31.7 (30.1-37.0)	<0.001

* Median (25-75 p); ** *Mann-Whitney U Test*

Table S4. Body weights of the experimental groups during the vaccination and immunization period (10-16 weeks). *

	Control groups				NAFLD groups			
	ND	HD2	HD3	P**	ND	HD2	HD3	P**
N	6	6	6		7	7	7	
Week 10	26.0 (25.6-27.9)	26.3 (25.2-29.3)	25.4 (24.3-27.0)	0.464	30.9 (29.6-31.8)	35.1 (31.4-37.2)	33.8 (29.9-37.0)	0.431
Week 12	27.4 (25.4-29.3)	25.7 (24.6-28.0)	26.0 (25.4-27.1)	0.512	32.5 (30.6-39.8)	37.5 (32.2-40.4)	33.8 (33.1-38.9)	0.498
Week 14	27.2 (25.3-29.3)	26.0 (25.2-27.6)	25.9 (25.3-26.9)	0.765	34.4 (31.6-42.7)	38.1 (36.4-44.2)	37.3 (35.4-37.7)	0.179
Week 16	27.4 (25.9-28.5)	26.4 (25.6-28.6)	26.0 (25.4-27.0)	0.370	34.4 (32.5-40.8)	42.5 (33.0-47.6)	37.2 (33.3-37.7)	0.530

*Median (25-75 p); ***Kruskal-Wallis* Test

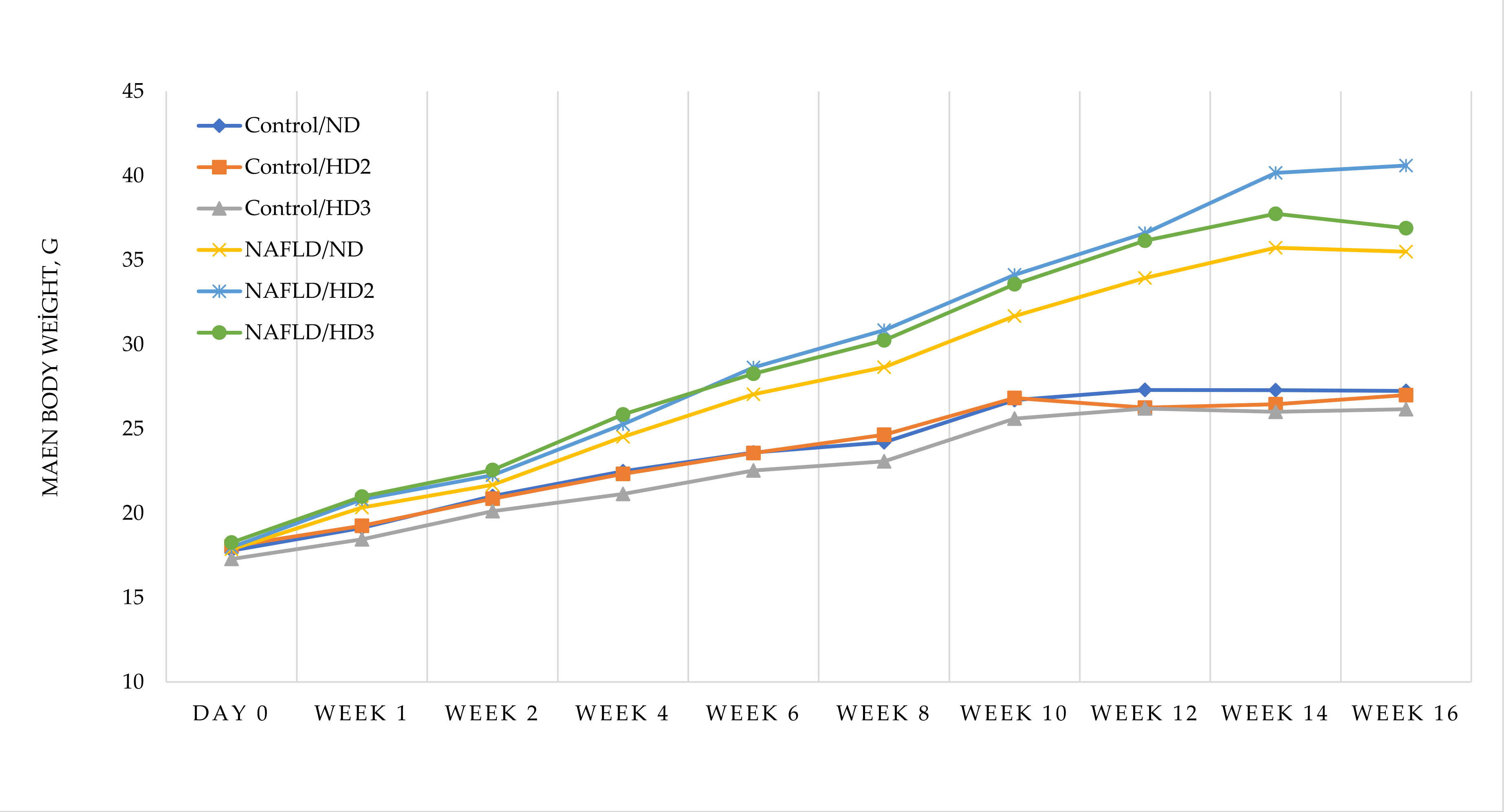

Suppl Figure 1. Body weight change by weeks in the experimental groups
